# Expert Elicitation for Latent Growth Curve Models: The Case of Posttraumatic Stress Symptoms Development in Children With Burn Injuries

**DOI:** 10.3389/fpsyg.2020.01197

**Published:** 2020-06-18

**Authors:** Duco Veen, Marthe R. Egberts, Nancy E. E. van Loey, Rens van de Schoot

**Affiliations:** ^1^Department of Methodology and Statistics, Utrecht University, Utrecht, Netherlands; ^2^Department of Clinical Psychology, Utrecht University, Utrecht, Netherlands; ^3^Association of Dutch Burn Centres, Beverwijk, Netherlands; ^4^Optentia Research Program, North-West University, Potchefstroom, South Africa

**Keywords:** Bayesian statistics, elicitation, expert judgment, expert knowledge, Latent Growth Curve Model, prior, prior-data (dis)agreement

## Abstract

Experts provide an alternative source of information to classical data collection methods such as surveys. They can provide additional insight into problems, supplement existing data, or provide insights when classical data collection is troublesome. In this paper, we explore the (dis)similarities between expert judgments and data collected by traditional data collection methods regarding the development of posttraumatic stress symptoms (PTSSs) in children with burn injuries. By means of an elicitation procedure, the experts’ domain expertise is formalized and represented in the form of probability distributions. The method is used to obtain beliefs from 14 experts, including nurses and psychologists. Those beliefs are contrasted with questionnaire data collected on the same issue. The individual and aggregated expert judgments are contrasted with the questionnaire data by means of Kullback–Leibler divergences. The aggregated judgments of the group that mainly includes psychologists resemble the questionnaire data more than almost all of the individual expert judgments.

## Introduction

Expert elicitation entails the extraction of information from experts and the translation of this information into a probabilistic representation. There are many reasons to elicit expert knowledge. In some cases, it is done to supplement existing data using priors that are informed by expert knowledge (van de Schoot et al., 2018). Alternatively, expert judgments allow for filling information gaps of certain data (Fischer et al., 2013; Dodd et al., 2017) or they can serve as a quality control for obtained data (Veen et al., 2018). Elicitation can also be used for forecasting purposes (Murphy and Winkler, 1974, 1984) or when there are no data available at all (Ho and Smith, 1997; Hald et al., 2016).

The use of expert knowledge is widespread across many disciplines. To give some examples, Dodd et al. (2017) elicited expert-based estimates for case-fatality ratios in HIV-positive children with tuberculosis who did not receive treatment; Barons et al. (2018) describe the use of expert judgments to create decision support systems with an example in food security; and Dewispelare et al. (1995) describe expert elicitation in relation to the long-term behavior of high-level nuclear waste repositories. For numerous other examples on elicitation practices, see for instance Chapter 10 of O’Hagan et al. (2006), listing applications in sales, medicine, nuclear industry, veterinary science, and many more. Other examples using a specific elicitation tool are given in Gosling (2018), while Cooke and Goossens (2008) describe a database of over 67,000 elicited judgments.

Recently, there is a growing interest in the use of expert elicitation in the social sciences. Where van de Schoot et al. (2017) only found two cases that reported the use of expert opinions to inform priors in 25 years of Bayesian statistics in psychology, this trend might slowly be changing. For instance, in their example related to a replication study in the field of psychology, Gronau et al. (2019) elicited expert judgments on effect sizes such that these could be used in informed Bayesian *t*-tests; Lek and van de Schoot (2018) elicited prior distribution from teachers concerning the math abilities of their students; and Zondervan-Zwijnenburg et al. (2017) elicited expert judgments on the correlation between cognitive potential and academic performance. Moreover, methods are being developed to facilitate expert elicitation in a flexible manner such that experts are guided in the elicitation process (Veen et al., 2017).

Whatever the reasons of the elicitation, the goal is to get an accurate representation of the experts’ beliefs and associated (un)certainty, which enables the representation of the experts’ domain knowledge in terms of a probability distribution. Overconfidence of experts is one of the crucial issues in expert elicitation (O’Hagan et al., 2006), resulting in elicited probability distributions with little uncertainty. In the seminal work of O’Hagan et al. (2006), feedback is named as the most natural way to improve the accuracy of elicited beliefs, with interactive software being almost essential for the effective use of feedback. This is corroborated by Goldstein and Rothschild (2014) who found that visual feedback can increase even laypeople’s intuitions about probability distributions. Over a decade has passed since the advice by O’Hagan et al. (2006), and many have followed it. Elicitation software can be split into more general and more customized variations. Some more general frameworks are, for instance, ElicitN, which was developed by Fisher et al. (2012) for the elicitation of count data. Truong et al. (2013) made a web-based tool for the elicitation of variogram estimates which describe a degree of spatial dependence. The elicitator was developed for indirect elicitation, creating a scenario-based elicitation (James et al., 2010; Low-Choy et al., 2012). Morris et al. (2014) developed MATCH which is based on the R package SHELF (Oakley, 2019) and which is a very general elicitation tool that allows multiple elicitation methods to be used interactively to elicit single parameters. Garthwaite et al. (2013) developed an elicitation procedure for generalized linear and piecewise-linear models. Runge et al. (2013) developed one for seismic-hazard analysis and Elfadaly and Garthwaite (2017) for eliciting Dirichlet and Gaussian copula prior distributions. Sometimes, more customized software is developed for specific elicitation settings (e.g., Bojke et al., 2010; Haakma et al., 2014; Hampson et al., 2014, 2015). To sum up, the use of software, customized or not, to increase the accuracy of the elicited beliefs is now common practice.

In this paper, we present an elicitation methodology especially designed for eliciting parameters of a Latent Growth Curve Model (LGM) regarding the development of posttraumatic stress symptoms (PTSSs) in children with burn injuries. LGMs are commonly used to analyze longitudinal data, especially in the social sciences (e.g., Buist et al., 2002; Catts et al., 2008; Orth et al., 2012). These models include repeated measurements of observed variables and allow researchers to examine change or development over time in the construct of interest. For extensive explanations of LGMs, see Duncan and Duncan (2004), Little (2013), and Little et al. (2006). Because in Western high-income countries, the incidence of severe burn injuries in school-aged children and adolescents is relatively low and obtaining a large enough sample to estimate LGMs is challenging. Nevertheless, to gain knowledge on the development of PTSSs in this group of children, these types of models are favored over simpler models. Expert elicitation might provide an alternative to data collection for cases like our motivating example where traditional data are sparse or they might supplement such data.

The main aim of this paper is to compare domain expertise expressed by experts in an elicitation setting to data on the same topic collected by means of traditional data collection methods (Egberts et al., 2018). Comparing experts’ domain knowledge to traditional data collection methods can provide unique insights into the topic of interest and the perception thereof. In the remainder of this paper, we first describe the methodology that is used to elicit the expert judgments. The methodology is an extension of the Five-Step Method (Veen et al., 2017) adapted to elicit multiple parameters. We elicit expert judgments from 14 experts, including nurses and psychologists working in the burn centers where data on PTSS in children were collected. Thereafter, we compare individual expert judgments to aggregated group-level expert judgments and data collected by means of traditional methods, followed by a reflection on the elicitation procedure. We conclude the paper with a *Discussion* section including recommendations for future research. All related materials for this study, including code and data, can be found on the Open Science Framework (OSF) website for this project at https://osf.io/y5evf/.

## Methods

In the first section, we describe the motivating example for this study. In the next section, we elaborate on the elicitation procedure and on software that has been developed. Finally, we describe the sample of experts (*N=14*) participating in the elicitation study. The study received ethical approval from our internal ethics committee of the Faculty of Social and Behavioral Sciences of Utrecht University. The letter of approval can be found in the data archive on the OSF website for this project.

### Motivating Example

The motivating example for this paper is the development of PTSS in children after a burn event. In a prospective study on child and parent adjustment after pediatric burns, data on these symptoms were collected in three Dutch and four Belgian burn centers. Children aged 8–18 years were eligible to participate in the study if they had been hospitalized for more than 24 h and if the percentage of total body surface area (TBSA) burned was at least 1%. A more detailed description of the overall study and sample can be found in Egberts et al. (2018). This sample consists of 100 children who reported on their symptoms of traumatic stress within the first month after the burn event (T1) and subsequently at 3 (T2) months post-burn. For the purpose of the current study, we also included the measurements obtained at 12 months (T3) post-burn. Children filled out the Children’s Responses to Trauma Inventory (CRTI, revised version; Alisic et al., 2006). This measure assesses four symptom clusters of posttraumatic stress, including intrusion (e.g., repetitive, intrusive recollections of the trauma), avoidance (e.g., avoiding conversations of the event), arousal (e.g., difficulty concentrating), and other child-specific responses (e.g., feelings of guilt). Further details on this measure can be found in Alisic et al. (2011).

**FIGURE 1:**
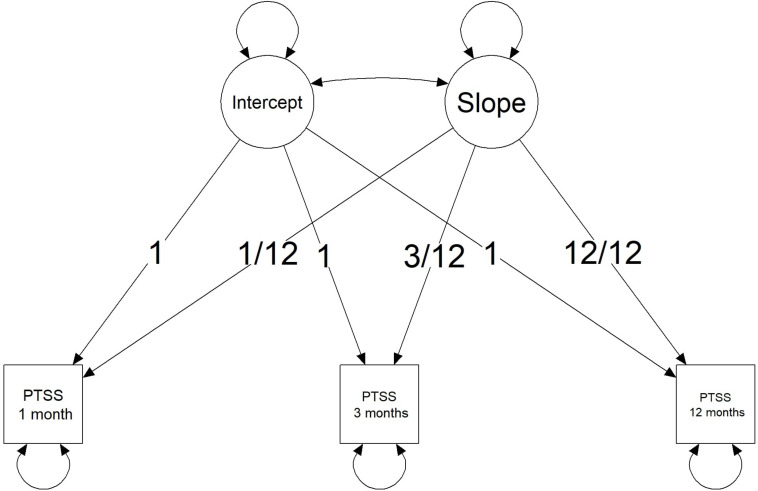
Visual representation of a Latent Growth Curve Model with three observed time points for posttraumatic stress symptoms (PTSSs).

As the current study includes three measurements of PTSS at different time points, a straightforward model to analyze the development of PTSS is an LGM. Figure 1 provides a visual representation of an LGM for this motivating example. The model is parameterized such that the latent intercept provides an estimate for PTSS in the first month after the burn event. The latent slope describes the change in PTSS at 1 year post-burn. Parameterizing the slope by year instead of per month is done to ease the reasoning in the elicitation procedure. Furthermore, the scale of the PTSS scores has been standardized for the data of the prospective study and for the elicitation study. The scores can fall between 0 and 100. A zero score means that none of the symptoms of any of the clusters of posttraumatic stress is present. A score of 100 means that all symptoms from all clusters are present to their maximum extent. A standardized cutoff value of 42 was used to indicate clinical relevance of symptoms and corresponds to the cutoff value provided in the CRTI manual. *Via* the OSF website for this project, supplementary materials can be found that describe the LGM analysis for these data, including assessment of the extent to which the LGM fits the data over the three time points.

### Expert Elicitation

To optimally prepare the experts within the limited time that was allocated for each elicitation, a short introduction was presented by the researchers conducting the elicitation (DV and ME), hereafter named the facilitators. The facilitators presented the experts with a brief overview of what expert elicitation is, what it can be used for, and how to interpret the probability distributions that are used to represent their beliefs. Thereafter, to familiarize the experts with the elicitation procedure itself, an example elicitation for an unrelated topic was presented to the experts using the same elicitation tool. After the example elicitation, the facilitators introduced the specifics related to the motivating example and the actual elicitation. Experts were instructed to think of the same reference population as used in the questionnaire study (i.e., children hospitalized for at least 24 h in one of the three Dutch or four Belgian burn centers with a minimum of 1% TBSA burned). Moreover, the CRTI symptom clusters were introduced, including specific examples of symptoms assessed with this measure. In addition, the measurement scale and research question were introduced, and experts were invited to ask questions to clarify any part of the procedure. Once the experts stated that they were ready to continue with the elicitation, they were requested to sign the informed consent letter, which they received prior to the elicitation. If they agreed, they also agreed to the recording of the elicitation procedure. The experts were requested to reason aloud during the elicitation. The recordings were transcribed to provide additional insights into the elicitation procedure and to track possible differences between experts. The experts carried out the elicitation procedure using the software that is described next.

The software and procedure in this study were based on the Five-Step Method developed by Veen et al. (2017), with a slight adaptation to elicit multiple parameters instead of a single parameter. The Five-Step Method decomposes the elicitation process in multiple smaller steps, providing visual feedback at each stage of the elicitation procedure. By decomposing the elicitation task and providing visual feedback, the procedure aims to reduce bias, for instance from overconfidence. The software has been developed in the form of a Shiny web application (Chang et al., 2019). Using Shiny to develop elicitation tools is not uncommon, see, for instance, Hampson et al. (2014), Hampson et al. (2015), and the original Five-Step Method by Veen et al. (2017). In what follows, we describe the Five-Step Method as implemented for this specific study for each expert. Note that steps 3 and 4 were repeated for each parameter.

**FIGURE 2:**
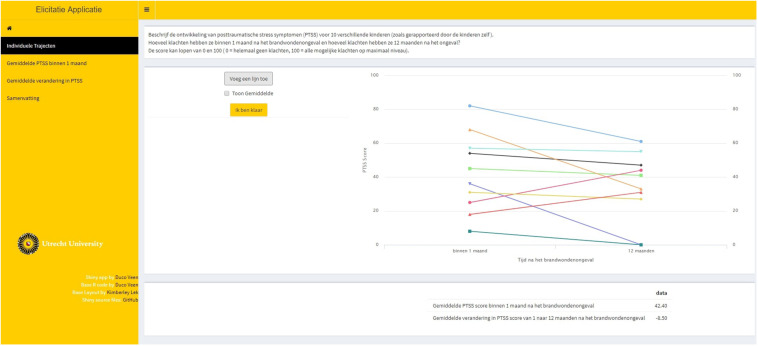
Step 1 of the elicitation procedure. Trajectories of posttraumatic stress symptom (PTSS) development were elicited for 10 individuals that are representative for the population. From these trajectories, point estimates for the average intercept and the average slope were obtained.

**Step 1.** Ten fictive individual PTSS trajectories were elicited for an LGM. These individual trajectories should be representative for the population. From these individual trajectories, we could deduce information on the point estimates for the average intercept and average slope parameters. This first step is called indirect elicitation because no statement is required directly concerning the parameters of interest. Figure 2 provides a visual representation of step 1.

**FIGURE 3:**
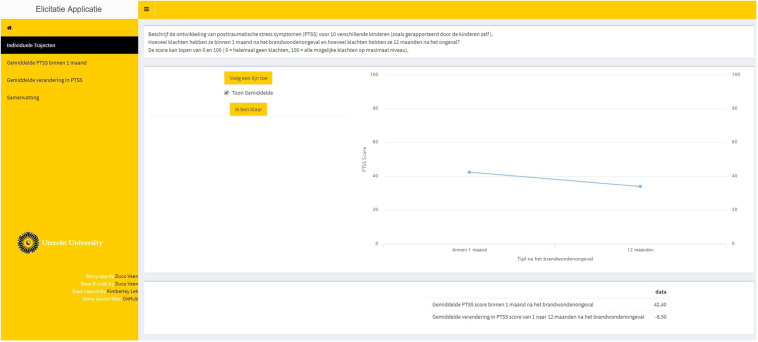
Step 2 in the elicitation procedure, providing visual feedback on the extracted average trajectory based upon the experts’ provided individual trajectories.

**Step 2.** Feedback was provided on the average trajectory that was based upon the 10 individual trajectories that the expert provided. The expert could accept this as the average trajectory and thereby accept point estimates for the average intercept and slope, or the expert could adjust his or her input in step 1. Figure 3 provides a visual representation of step 2.

**Step 3.** The experts provided a reasonable lowerbound and upperbound for the point estimates of the group mean intercept and the group mean slope that were obtained using steps 1 and 2. The lowerbound and upperbound were used to determine the scale and shape of the probability distribution that was used to represent the experts’ beliefs. This is called direct elicitation because the experts provided information directly related to the parameters of interest.

**FIGURE 4:**
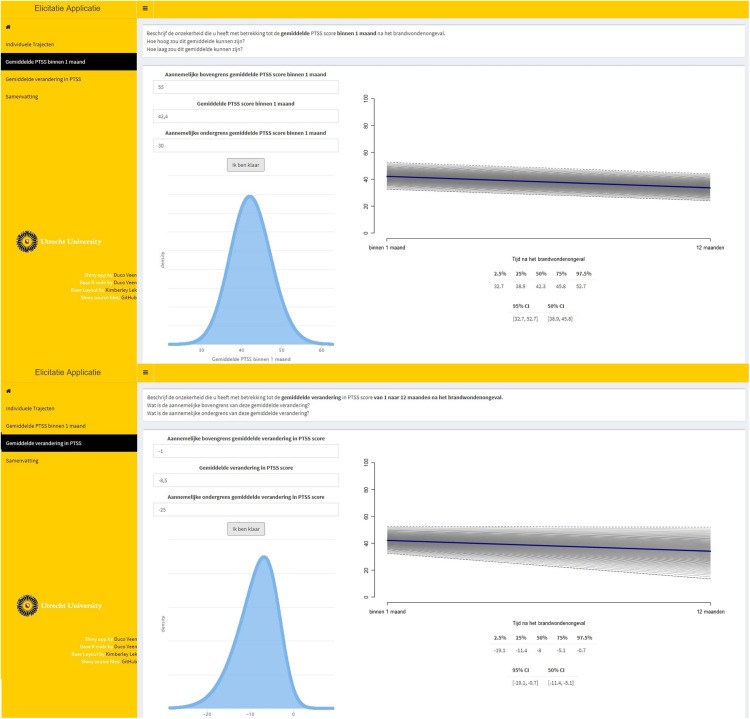
Steps 3 and 4 of elicitation procedure for the average intercept, top panel, and the average slope, bottom panel. The input that was required for step 3 was provided in the fields on the top left of the tab in the elicitation software. The single-parameter feedback was provided on the bottom left of the tab, displaying the fitted prior density with respect to that parameter. The effect on the implied average trajectory was displayed on the right-hand side of the tab. The average trajectory that was accepted in step 2 is displayed, and a gray band has been added around this average trajectory that represents the 95% credible interval (CI) for the average trajectory. In the top panel, only the uncertainty with respect to the intercept was added to the average trajectory. In the bottom panel, the uncertainty with respect to both the intercept and the slope was added.

**Step 4.** Feedback was provided on the probability distribution that was used to represent the experts’ beliefs. Figure 4 provides a visual representation of steps 3 and 4 with respect to the average intercept, top panel, and the average slope, bottom panel. Single-parameter feedback was provided in the form of a prior density plot, as well as the effect on the implied average trajectory. The experts could accept and confirm the representation of their beliefs or adjust their input in step 3.

**FIGURE 5:**
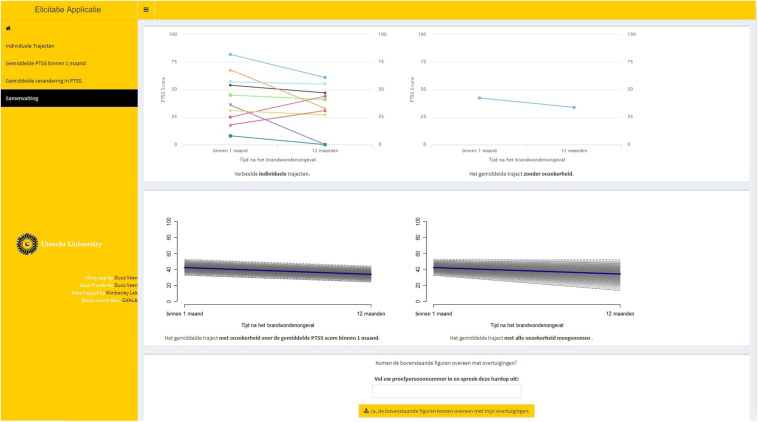
Summary page of the elicitation procedure. The top left plot within the page displays all individual trajectories that the expert specified. The top right plot displays the average trajectory that was obtained based on those individual trajectories. The bottom left plot displays the average trajectory with uncertainty (95% CI) concerning the intercept value taken into account. The bottom right plot displays the average trajectory with uncertainty (95% CI) concerning both the intercept value and the slope value taken into account.

**Step 5.** The experts were shown a summary page on the elicitation, see Figure 5. If the experts accepted the representation of their beliefs, the probability distributions were now ready to be saved and used in the analyses.

### Sample of Experts

Fourteen experts from all three Dutch burn centers participated in the elicitation study. These experts had different professions, including (child) psychologists, pediatric nurses, specialized nurses for burn injuries, and nurses with an additional master’s degree [master of science (MSc)]. During the process of obtaining this degree, these nurses worked closely with psychologists and observed their work. Though they are employed at the same burn centers, the tasks and expertise of nurses and psychologists differ: nurses are assumed to have a broader clinical view, taking into account physical and psychological aspects of adjustment, but not necessarily PTSS. Psychologists have a more focused clinical view and have specific expertise on PTSS after traumatic events. Because reporting the individual expert professions would remove almost all anonymity, we ensured that no elicited probability distributions can be associated with individual experts and therefore categorized the experts into two groups. The first group consisted of experts who have obtained an MSc degree (*N=7*), and the second group consisted of experts who have not (*N=7*). As the first group consisted mostly of psychologists or experts with at least some education in psychology, we shall refer to this group as the psychologists. The second group consisted mostly of nurses with a variety of additional specializations, and we shall refer to this group as the nurses. The two groups are considered large enough for elicitation studies. Cooke and Goossens (1999) recommend to use the largest possible number of experts, stating that four is the minimum. We were able to include seven experts in both groups of experts.

## Results

This section first covers a descriptive part on the expert judgments. We report the priors that the experts provided and the mixture priors that can be made from these expert judgments on an aggregated and group distinct level. Thereafter, we report prior-data (dis)agreement measures for all individual expert judgments and the mixture distributions. These prior-data (dis)agreements are based upon the data that were collected in the prospective study by Egberts et al. (2018). Finally, we report notable results from the audio recordings. Note that the quantitative results, analyses, and an overview of individual expert judgments can be found *via* the OSF website for this project at https://osf.io/y5evf/. The transcripts of the audio recordings include many identifying characteristics with respect to both the experts and patients they described during the elicitation and to preserve privacy, so these are not available. This is in accordance with the ethical approval agreement.

### Individual and Group Expert Judgments

**FIGURE 6:**
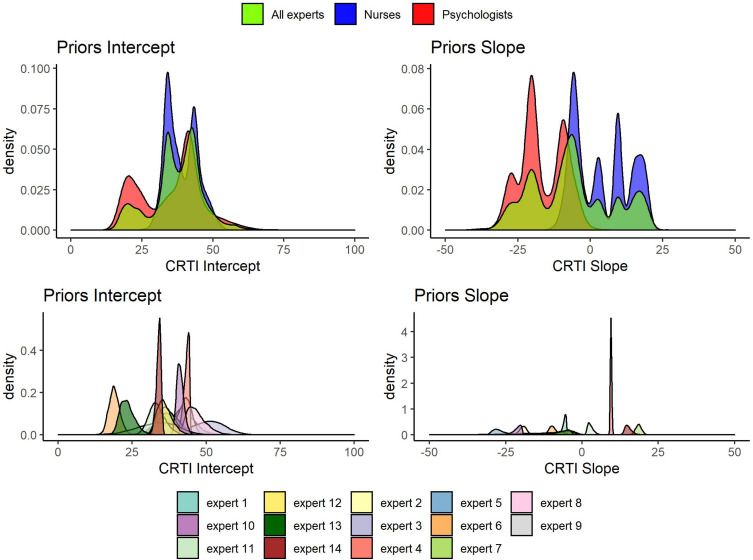
Elicited prior densities from all experts and the associated mixture priors for all experts, the psychologists’ group, and the nurses’ group regarding both the mean intercept and the mean slope of posttraumatic stress symptom (PTSS) development.

**FIGURE 7:**
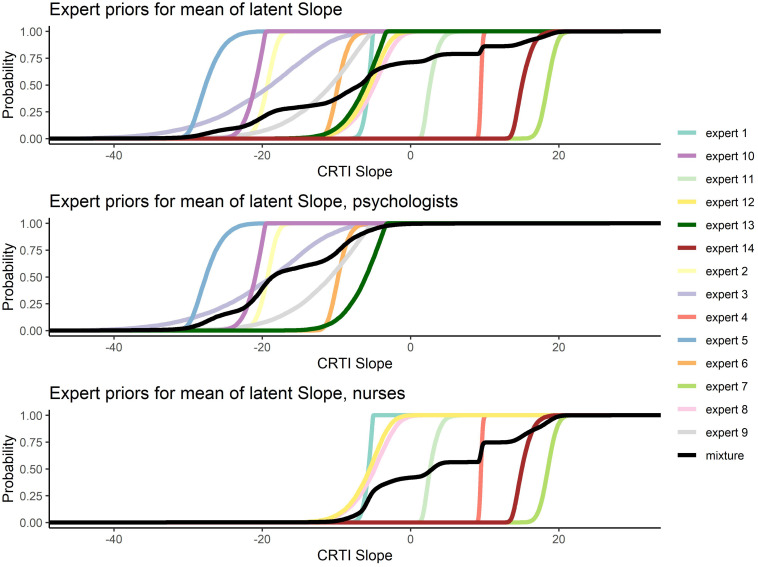
Elicited prior distributions from all experts and the associated mixture priors for all experts, the psychologists’ group, and the nurses’ group regarding the mean slope of posttraumatic stress symptom (PTSS) development. The cumulative distributions are presented. There was a notable difference in expert judgments between the psychologists’ and the nurses’ groups.

All 14 expert judgments had been elicited, allowing them to specify a skewed normal distribution parameterized according to Burkner (2019). In Figure 6, all the elicited individual expert prior densities can be found as well as the mixture density for all experts, the psychologists’ group and the nurses’ group regarding both the mean intercept and the mean slope of PTSS development^1^. Figure 6 shows that the expert judgments differed quite substantially. Especially concerning the development of PTSS as expressed by the slope parameter, we can see that experts disagreed on the direction of the effect and with a lot of confidence. When we look at the groups of experts, an interesting pattern emerges. If we combine the expert judgments of the psychologists and the nurses into their respective group, the nurses turn out to have a substantially different view from the psychologists. Not only did the nurses’ judgments express on average a higher initial amount of PTSS in the population, their combined view also expressed that these initial PTSS scores are quite likely to increase on average over time. The psychologists in contrast assigned almost no probability to an increase in the average PTSS score over the time period of a year; see Figure 7 for a closer look.

### Prior-Data (Dis)Agreement

**FIGURE 8:**
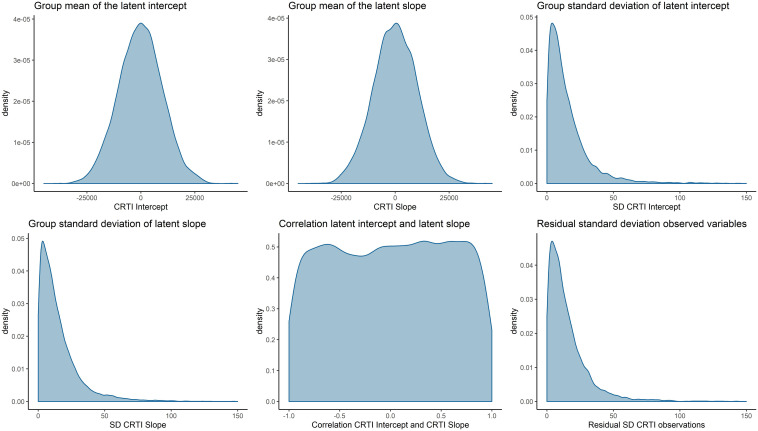
Visual representation of the prior densities that are used to obtain the reference posterior. The prior densities are α_1_∼*N*(0,10^8^), α_2_∼*N*(0,10^8^), ψ_11_∼*half*−*t*(3,0,196), ψ_22_∼*half*−*t*(3,0,196), ψ_21_∼*U*(−1,1), and θ∼*half*−*t*(3,0,196).

**FIGURE 9:**
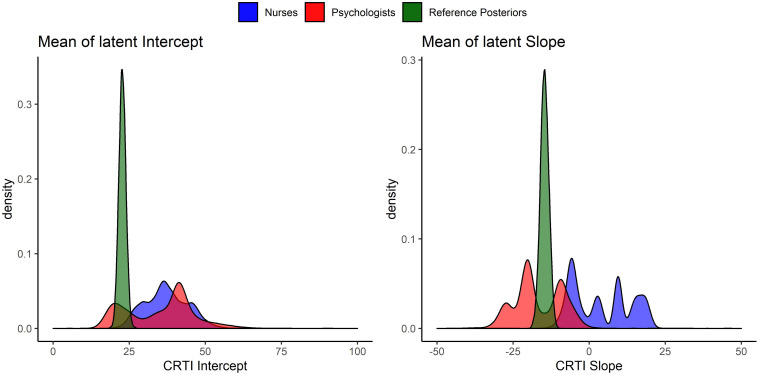
Visual representation of the reference posterior densities for the group mean of latent intercept and slope with the group expert priors for the parameters. The reference posteriors are approximately distributed, *CRTI*_*Intercept*_∼*N*(22.7,1.3), and *CRTI*_*Slope*_∼*N*(−14.6,1.9).

To assess the (dis)agreement of experts’ judgments with the data from the prospective study by Egberts et al. (2018), we used Kullback–Leibler (KL) divergences (Kullback and Leibler, 1951) between the posterior distribution that is based upon the data and an uninformative benchmark prior as well as the individual and aggregated expert judgments. Using information theoretical distance measures to asses prior-data (dis)agreement in this manner has previously been discussed by, for instance, Bousquet (2008), Lek and van de Schoot (2019), and Veen et al. (2018). KL divergences provide us with an indication of how much information is lost as we approximate distribution π_*1*_ by another distribution π_*2*_. A higher divergence indicates a higher loss of information. In this case, π_*1*_ will be the posterior distribution based upon the data and an uninformative benchmark prior, to which we refer as the reference posterior. We approximate the reference posterior with the elicited prior distributions and report the loss of information. For an overview of the priors that are used to compute the reference posterior, see Figure 8. Figure 9 visualizes the reference posteriors for the group mean latent intercept and slope. We used the uninformative benchmark 2 priors that are described in the next paragraph. The differences are negligible with the use of benchmark 1 priors, as can be seen in the supplementary materials that describe the LGM analysis. This demonstrates the principle of stable estimation; the priors are overwhelmed by the data.

In addition to comparing the expert priors to the benchmark posterior, we added two other comparisons to create a frame of reference. Two benchmark situations are added, and their loss of information is calculated. In the situation of benchmark 1, we would take some information regarding the measurement instrument into account. The scale of the measurement instrument was standardized such that values are between 0 and 100; therefore, a *U*(0,100) prior on the group mean intercept would cover all possible parameter values. With the parameterization such that the final time measurement implies a change of 1 times the individual latent slope parameter, taking the standardized scale into account, a *U*(−100,100) prior on the latent slope covers all possible parameter values and declares them equally possible. For benchmark 2, we take two *N*(0,10^8^) priors on the latent group mean intercept and slope. It is still common practice, when using Bayesian statistics, to rely on default or uninformative priors when calculating posterior distributions. For instance, in Mplus, the default priors for these specific parameters are *N*(0,∞) (Asparouhov and Muthén, 2010, Appendix A), which are used in, for instance, McNeish (2016), and van de Schoot et al. (2015). Lynch (2007, chapter 9), using precision instead of variance, specifies *N*(0, 0.0001) priors for these parameters. Benchmark 2 reflects this practice.

The KL divergences are reported in Table 1 and are the numerical representation of the loss of information that occurs by approximating the reference posterior densities from Figure 9 by the densities that can be seen in Figure 6 for the experts’ priors. It seems that most experts are in disagreement with the collected data from Egberts et al. (2018). There are some individual exceptions, notably experts 9 and 13, who have a view that is very similar to the collected data, while some experts provide a similar view with respect to one of the two parameters, e.g., experts 3 and 6. It is notable that the group of psychologists in particular and the group of experts as a whole show less loss of information with respect to the data than most experts on both parameters. Finally, what is noteworthy is that benchmark 1, which has no preference for any part of the parameter space covered by the measurement instrument, resembles the data more than most expert judgments and more than the nurses’ judgments as a group.

**TABLE 1:** Kullback–Leibler divergences for all individual and mixture priors to the reference posterior.

	Intercept	Slope
Benchmark 1	3.04	3.56
Benchmark 2	8.56	8.39
Nurses	8.19	5.88
Psychologists	1.99	2.18
All	2.72	2.63
Expert 1	42.87	59.18
Expert 2	45.16	25.87
Expert 3	6.71	1.23
Expert 4	72.86	55.38
Expert 5	5.66	98.32
Expert 6	2.10	22.17
Expert 7	79.20	59.61
Expert 8	46.97	4.37
Expert 9	2.48	1.28
Expert 10	43.74	67.55
Expert 11	12.78	64.56
Expert 12	99.94	4.88
Expert 13	0.35	3.62
Expert 14	75.00	74.11

### Audio Recordings

The following observations were noteworthy in the transcripts of the audio recordings. All psychologists referred specifically to the concept of PTSS during the elicitation procedure. The group of nurses mentioned stress a lot, but only two nurses actually referred to PTSS specifically. Three psychologists reflected on the linearity assumption of the model and noted that non-linear trajectories often occur. Five of the nurses expressed sentiments that the more severe cases came to mind more easily and therefore might be overrepresented in their beliefs. Only one psychologist expressed a similar statement. Three experts, one psychologist and two nurses, actively reflected on the visual feedback and adjusted their input in the elicitation tool based on this. One expert, a nurse, stated that although he or she was sure about the direction of the trajectory, he or she felt unsure about the associated numerical representation. Finally, one expert, a nurse, repeatedly mentioned that he or she found the task hard to do.

## Discussion

We were able to elicit expert judgments with respect to the development of PTSS in young burn victims from 14 experts and contrasted this with data collected in a traditional way by means of a questionnaire. Our study demonstrates differences in views between experts. On an individual basis, the experts were particularly in disagreement with regard to the change of PTSS at 1 year post-burn. There is little overlap in expert beliefs when we look at the elicited prior densities for the slope parameter. The expert judgments not only differed from one individual to the next, but there also seems to be a relationship between the experts’ role in the post-burn treatment process and their view on the children’s development of PTSS. The two groups of experts differed notably in the aggregated elicited judgments: aggregated judgments of the psychologists seemed to align with the data collected by Egberts et al. (2018) while the nurses’ judgments seemed to differ more.

With respect to the differences between the two groups of experts, the most remarkable difference was found with respect to the slope parameter. The aggregated views of the groups of experts result in distributions with more uncertainty compared to the individual experts’ beliefs. The dispersed views of the experts put together ensure coverage of a larger part of the parameters space than the individual expert judgments do. Interestingly, the more uncertain distributions still clearly present a difference in views regarding the development of PTSS in young burn victims between the nurses’ expert group on the one hand and the psychologists’ expert group and the data collected by Egberts et al. (2018) on the other. The aggregated judgments from the psychologists assigned almost no probability to the group average PTSS increasing at 1 year post-burn. The aggregated judgments from the nurses, in contrast, assigned a lot of probability to an increase of the group average PTSS at 1 year post-burn. As there is no grounded truth, we cannot conclude which views are a better, or worse representation. However, the results do indicate that the nurses and the psychologists are not in agreement on what happens with respect to the development of PTSS in young burn victims, despite having received similar information about (assessment of) PTSS prior to the elicitation.

The audio recordings of the elicitation settings provide a possible explanation for this important distinction. All psychologists at some point during the elicitation referred to, or specifically mentioned, the construct of PTSS. The group of nurses mentioned several sources of distress, but only two nurses actually referred to PTSS, while one of them judged the 1-year post-burn PTSS to decrease. As burn victims can indeed experience other sources of distress, e.g., related to the development of scar tissue or operations they have to undergo, nurses may have convoluted PTSS with other patient symptoms. This could also explain why the aggregated nurses’ view judged the initial PTSS level to be higher for the group average than the aggregated psychologists’ view. Overall, the differences possibly reflect the fact that psychologists are trained to diagnose and treat PTSS, whereas nurses are primarily concerned with procedural and physical care for the patient and are not involved in diagnosing and treating PTSS. In a future study, it could be of interest to investigate the experts’ knowledge of the constructs of PTSS and see if this is predictive of KL divergence.

Besides differences between the nurses and the psychologists, we also found a substantial difference between the reference posteriors that provided a representation of the data from Egberts et al. (2018) and the aggregated nurses prior. In Figure 9, it can be seen that the psychologists’ views overlapped with the reference posteriors. The nurses’ views, however, showed almost no overlap with reference posteriors. This could also be assessed numerically, as was done with the KL divergences in Table 1. Because the aggregated nurses prior had little overlap with the reference posteriors, the Benchmark 1 priors, i.e., uniform priors that take the information of the measurement instrument into account, outperformed this group in terms of loss of information. This implies that the data collected by Egberts et al. (2018) were better approximated by an uninformed expression of the questionnaire’s measurement properties than by the nurses’ group prior. The children in the study by Egberts et al. (2018) expressed a lower quantity of PTSS in their self-reported questionnaires compared to the nurses’ expert judgments on PTSS for this population.

There can be several explanations for this discrepancy. First, the questionnaire may have resulted in underreporting of symptoms, a view also expressed by one of the experts. In line with this, Egberts et al. (2018) found that mothers gave higher ratings of their child’s PTSS compared to the children themselves. On the other hand, mothers’ ratings appeared to be influenced by their own symptoms of posttraumatic stress and fathers did not report higher ratings of PTSS compared to their children. Alternatively, the discrepancy could be explained by the elicitation of the expert judgments. Especially the nurses’ group reported higher PTSS levels compared to the self-reports, and the previously mentioned convolution of symptoms and lack of specific knowledge about PTSS might be a cause for this observation. In the recordings of the elicitation settings, we found another possible cause. Five of the nurses expressed sentiments that the more severe cases came to mind more easily and therefore might be overrepresented in their beliefs. This is a clear expression of the well-known availability heuristic (Tversky and Kahneman, 1973) that can cause biases in elicitation studies (O’Hagan et al., 2006). In the psychologists’ group, only a single expert expressed a similar remark. The availability heuristic, if not remedied, might cause the discrepancy between the reference posteriors and the expert judgments.

The study showed that providing visual feedback on the representation of the experts’ beliefs can lead to experts adjusting their input such that obvious incorrect representations of their beliefs are remedied. Unfortunately, it is not possible to validate whether the representation of the experts’ beliefs actually corresponds to the “true” beliefs of the expert (O’Hagan et al., 2006; Colson and Cooke, 2018). However, one of the main reasons to use elicitation software is to ameliorate the effects of heuristics and biases by getting experts to actively reflect on the probability distribution that will be used to represent their beliefs. In the recordings, three experts actively reflect on their distributions, adjusting them based on the visual feedback. For this purpose, the elicitation software seems to have worked well. Nevertheless, it seems from our current study that even with the graphical feedback, some experts might still suffer from overconfidence. Expert 11, for instance, stated *“*… *of course, I have a lot of uncertainty anyway.”* However, this does not seem to be reflected in the elicited distribution which has a 99% CI for the latent intercept (27.2, 41.7) and the latent slope (1.2, 5.9). As the experts were only available to us for a limited time, we did not provide a specialized training aimed at elicitation and overcoming heuristics associated with elicitation tasks, which might be a limitation for the current study and the associated (individual-level) results.

This study indicates that aggregating expert judgments could potentially mitigate the severity of individual biases, as one has to rely less on single, possibly overconfident, experts. The aggregation of all experts’ judgments or of only the psychologists’ judgments leads to less discrepancy between the traditionally collected data and the elicited beliefs in comparison to almost any individual expert and the benchmarks. Aggregating or pooling of expert judgments into a single distribution is common in elicitation studies and can be done in several manners. In our current study, we used opinion pooling with equal weights (O’Hagan et al., 2006, Chapter 9). Alternatively, there is much literature on how expert judgments could be weighted in the aggregation of views. The classical model (Cooke, 1991, Chapter 12) is one of the foremost examples of this. In the classical approach, calibration questions are used to assess the experts. Based on the calibration questions, experts’ judgments on the target question or question of interest are weighted to together form the groups’ weighted prior beliefs. The calibration questions should be related to the question of interest, and their answers should be known but not to the experts (Colson and Cooke, 2018). It is recommended to have at least eight to 10 calibration questions if dealing with continuous variables (Cooke, 1991, Chapter 12). The experts are elicited concerning the question of interest and the calibration questions. Their answers on the calibration questions are evaluated against the known true values, and the experts are rated on their informativeness and accuracy (Cooke, 1991; Colson and Cooke, 2018). The ratings of the weighting components are based upon the idea of KL divergences (O’Hagan et al., 2006, Chapter 9) such as we used to compare the experts’ judgments against the collected data on the question of interest directly. As far as we know, there have not been any studies using the classical approach in the social sciences. Finding calibration questions turns out to be a hard problem, as knowing the true answer to these questions is required. We described the KL divergence between the target question and the experts’ judgments, but calibrating experts based on these weight components would be putting emphasis on the traditionally collected data twice. As the traditionally collected data might suffer from biases too, consider for instance the total survey error framework (Groves et al., 2011, Chapter 2) including non-response error and measurement error, this double emphasis might not be desirable. Instead, our equal weights aggregation approach relied on the inclusion of experts with balance in views and diversity in backgrounds (Cooke and Goossens, 1999).

In conclusion, it is possible to express the experts’ domain knowledge as prior distributions using the described methodology and compare these elicited distributions to traditionally collected data. The individual expert judgments in general show quite some discrepancy in comparison to traditionally collected data, although there are notable exceptions to this. When considering the mixtures of the groups of experts, the discrepancy becomes less pronounced, especially for the psychologists’ group. The psychologists’ mixture prior has less KL divergence than mostly any individual expert and notably less KL divergence than Benchmark 1, the uniform prior that takes the information of the measurement instrument into account. The expert judgments add information to the research area, and exploring (dis)similarities between expert judgments and traditional data opens up two exciting avenues for future research. One being the collection of data on the experts that might be predictive for the amount of KL divergence they exhibit with respect to traditionally collected data. The second avenue is the organization of a Delphi-like setting with all experts after the individual judgments are collected and compared with traditional data. The group setting can provide insights into the reasons behind the discrepancies between traditional collected data, individual experts, and groups of experts. If done in a longitudinal manner, this could start a learning cycle in which data and experts converge. Predicting and explaining (dis)similarities between experts’ judgments and traditional data such as results of questionnaires can be a potential new line of research for the social sciences.

## Data Availability Statement

All related materials for this study, including code and data, can be found on the Open Science Framework (OSF) web page for this project at https://osf.io/y5evf/. The transcripts of the audio recordings include many identifying characteristics with respect to both the experts and patients they described during the elicitation and to preserve privacy, so these are not available.

## Ethics Statement

The studies involving human participants were reviewed and approved by Ethics Committee of the Faculty of Social and Behavioral Sciences of Utrecht University. The patients/participants provided their written informed consent to participate in this study.

## Author Contributions

All authors have been involved in the design of the study and the elicitation procedure. DV programmed the elicitation software. ME arranged the elicitation meetings with the experts. DV and ME conducted all elicitation procedures together. DV wrote and revised the manuscript with contributions and feedback provided by ME, NL, and RS.

## Conflict of Interest

The authors declare that the research was conducted in the absence of any commercial or financial relationships that could be construed as a potential conflict of interest.

## Footnotes

1 Note that the mixtures are based on normal approximations of the elicited skewed normal distributions due to computational instability of the mixture distributions when skewed normal expert priors were used. All experts are weighted equally in the mixture for all experts. The mixture distributions of the nurses and psychologists can be seen as a special case of weighting in which half of the experts receive a weight of 0 and the other half are equally weighted.
